# Anatomical Analysis of Bicipital Groove and Its Spur Formation Using 3D-CT: A Retrospective Observational Study

**DOI:** 10.3390/life14121529

**Published:** 2024-11-21

**Authors:** Hyun Seok Song, Hyungsuk Kim

**Affiliations:** Department of Orthopedic Surgery, Eunpyeong St Mary’s Hospital, School of Medicine, The Catholic University of Korea, Seoul 03312, Republic of Korea; hssongmd@hanmail.net

**Keywords:** rotator cuff tear, biceps brachii, spur, computed tomography, 3D imaging

## Abstract

Concomitant long head of biceps (LHB) pathologies commonly occur with rotator cuff tears, but LHB problems are often underestimated. There is a lack of studies on the correlation between bicipital groove morphology and biceps pathology, as well as the significance of bony spurs around the groove. This study analyzed the anatomical parameters of the bicipital groove and spur formation using 3D-CT in 111 patients. Biceps spurs were evaluated using 3D and 2D-CT images, and patients were grouped by age (below and above 55 years). The detection rate of biceps spur was higher with 3D-CT (54.0%) than with 2D-CT (42.3%). Spur incidence was significantly greater in older patients (60.3% vs. 45.8%). The average groove width was narrower in older adults (*p* = 0.006) and larger in men compared to women (*p* = 0.000). The average spur height was also greater in men (*p* = 0.039). Lateral spurs were the most common type that was observed. This study highlights the differences in bicipital groove morphology and spur formation by age and gender, with 3D-CT being more effective in detecting biceps spurs.

## 1. Introduction

Pathologies of the long head of biceps (LHB) tendon, such as tendinitis, tenosynovitis, subluxation, dislocation, and tears, are common causes of anterior shoulder pain [1,2,3,4,5]. These conditions frequently present with symptoms such as pain, weakness, and clicking sensations during arm movements [6,7]. Tendinitis and tenosynovitis are associated with overuse or degenerative changes, whereas subluxation and dislocation result from instability of the biceps tendon within the intertubercular groove [7,8,9].

Magnetic resonance imaging (MRI) studies of painful shoulders have reported an LHB tendon pathology prevalence of up to 66% [10]. Furthermore, LHB pathology often coexists with rotator cuff tears, with a concomitant occurrence rate of approximately 70% [1,5,11,12,13]. Despite this high prevalence, LHB tendon issues are frequently underestimated and, if not adequately addressed, can significantly impair shoulder function and reduce quality of life.

If an LHB lesion is overlooked during arthroscopic rotator cuff repair, patients may experience persistent postoperative pain [14,15]. Thus, a preoperative diagnosis and a comprehensive treatment strategy for LHB pathology are essential. However, distinguishing LHB lesions from rotator cuff pathology based solely on symptoms and physical examinations can be challenging [16]. The Speed’s and Yergason’s tests, commonly used for detecting LHB pathology, have shown relatively low sensitivity (67% and 37%, respectively) and specificity (55% and 86%, respectively) [6]. Furthermore, Mohtadi et al. reported a low agreement rate (60.4%) and a low concordance rate (37.7%) between preoperative MRI findings and arthroscopic findings of LHB pathology [10]. Despite the use of physical examinations and imaging studies, accurately identifying LHB pathology before surgery remains challenging.

Previous studies on the morphology of the bicipital groove, including the width and depth of the intertubercular groove and the angle of the medial wall, have proposed certain hypotheses [7,17]. Specifically, it has been suggested that a narrower bicipital groove may be associated with a higher incidence of LHB wear, while a smaller medial wall angle and shallower groove depth could be linked to LHB instability [17]. However, there is a lack of studies proving the correlation with bicipital groove morphology and biceps pathology.

Bony spurs around the bicipital groove were first identified in imaging studies using simple radiographs and ultrasonography [8,17]. Cone et al. [7] described medial wall and groove spurs of the bicipital groove in a cadaveric study, suggesting that these osseous protrusions could contribute to LHB tears. Ueberham et al. [9] identified a “critical zone” within the intertubercular sulcus of the humerus, where the long head of the biceps tendon tends to wear down, reporting lateral spurs in 32% and medial spurs in 23% of cases in a cadaveric study. Despite these findings, spurs around the bicipital groove have received less attention compared to acromial spurs in shoulder pathology [18]. To our knowledge, there is a shortage of studies specifically addressing bicipital groove spurs.

The purpose of this study was to analyze the incidence of bicipital spurs based on gender and age using three-dimensional (3D) computerized tomography (CT) scans. Specifically, we aimed to assess both medial and lateral spurs of the bicipital groove. Additionally, we examined the anthropometric parameters of the bicipital groove in an Asian population. Finally, we compared the detection rate of bicipital spurs on 2D axial CT with that on 3D reconstructed images. Our hypothesis was that anthropometric differences would be observed between genders and age groups, and that the detection of bicipital spurs would be lower on 2D axial CT compared to 3D reconstructed images.

## 2. Materials and Methods

### 2.1. Patient Enrollement

A total of 111 patients who underwent shoulder 3D-CT scans due to pathology around the shoulder joint at one tertiary hospital were enrolled in this study. Patients with proximal humeral fractures involving the bicipital groove, a history of prior trauma or surgery around the bicipital groove, or a history of surgery for biceps pathology were excluded. The mean age of the participants was 55.4 years (ranging from 44 to 84 years). To facilitate comparison based on age, we used the mean age of 55 years as a threshold to categorize the participants into two groups: Group I (under 55 years old) with 48 patients; and Group II (55 years and older) with 63 patients.

The study protocol was approved by our Institutional Review Board (PC13RISI0052). Given the retrospective nature of the study, the requirement for informed consent was waived.

### 2.2. Bicipital Groove Spur Observation on 3D CT

**Figure 1:**
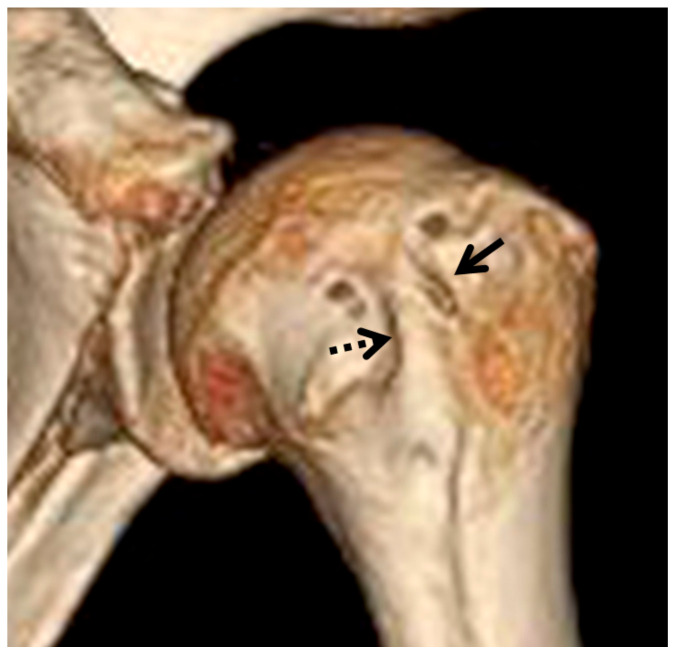
The three-dimensional CT image shows spurs around the bicipital groove. The solid arrow indicates a lateral spur, while the dashed arrow indicates a medial spur.

Acute-angled bony ridges or outgrowing spurs at the edge of the groove were recorded as bicipital spurs using both 3D reconstructed images and axial images from 2D-CT (Figure 1). The detection rates between the 3D reconstructed images and 2D-CT axial images were compared.

In the 3D reconstructed images, bony excrescences forming on the medial lip of the intertubercular sulcus were classified as medial wall spurs, while excrescences within the bottom half of the sulcus were categorized as groove spurs [7]. Additionally, lateral wall spurs of the bicipital groove were identified on the lateral lip of the sulcus. Cases where both medial and lateral spurs were observed were recorded as both spurs. The height of each spur was measured using axial images from 2D-CT.

### 2.3. Bicipital Groove Anthropometry

**Figure 2:**
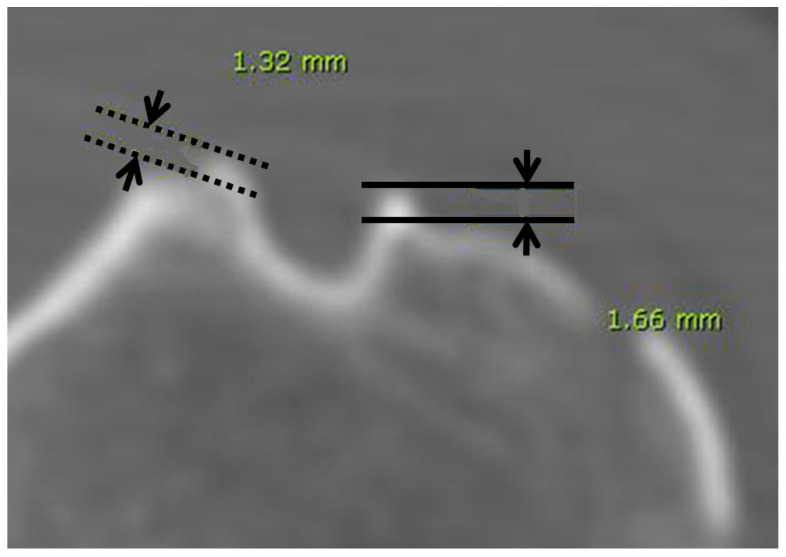
The axial CT image shows the bicipital spurs. The solid lines indicate the height of the lateral spur, while the dashed lines indicate the height of the medial spur.

**Figure 3:**
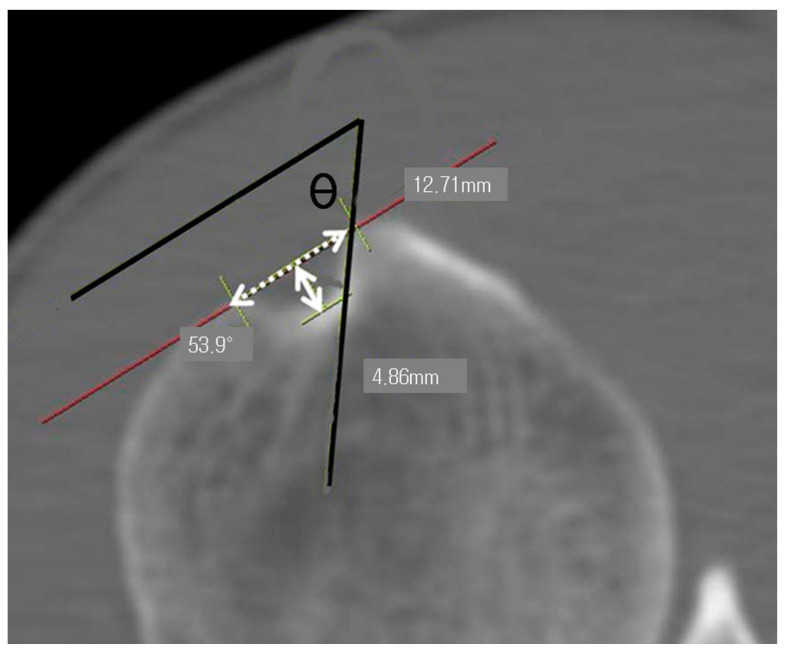
The axial CT image shows the parameters of the bicipital groove. The solid arrow indicates the depth, while the dashed arrow indicates the width of the groove. The angle (θ) represents the medial wall angle, formed by two solid lines: one along the medial wall and the other parallel to the line connecting the highest points of the medial and lateral walls.

Using 2D-CT axial images, the width of the bicipital groove was defined as the distance between the medial and lateral lips of the groove (Figure 2) [16]. The depth was measured as the vertical distance from the line connecting the medial and lateral lips (the intertubercular line) to the lowest point of the groove. To measure the medial wall angle, a line was drawn tangent to the medial wall of the bicipital groove (line of the medial wall), and the angle formed by the intersection of this line with the intertubercular line was recorded. All measurements, including the width, were taken at the narrowest part of the groove as observed in the axial view (Figure 3).

All measurements were conducted by two orthopedic surgeons (H.S.S. and H.K.) with over 10 years of experience in shoulder surgery. Interobserver reliability was assessed using intraclass correlation coefficients (ICC).

### 2.4. Statistical Analysis

A statistical analysis was conducted using IBM-SPSS Statistics version 24.0 (SPSS Inc., Chicago, IL, USA). For the analysis of the width, depth, and medial wall angle, the Student’s *t*-test (parametric) and Mann–Whitney test (non-parametric) were applied. The Chi-square test (parametric, one-way, single measures) and Fisher’s exact test (non-parametric, one-way, single measures) were used to analyze the presence of spurs. All statistical testing was performed with the level of significance set at 95% significance.

## 3. Results

### 3.1. Detection and Incidence of Bicipital Groove Spur

Bicipital spurs were observed in 60 cases (54%) using 3D reconstructed images and in 47 cases (42.3%) on axial images from 2D-CT. Notably, 20.3% of spurs detected on 3D reconstructed images were not observed on 2D-CT axial images.

The incidence of bicipital spurs on 3D reconstructed images according to age group was 45.8% (22 cases) in Group I (below 55 years) and 60.3% (38 cases) in Group II (over 55 years). Regarding gender, the incidence was 59.3% (35 cases) in men and 48.1% (25 cases) in women. A statistically significant difference in incidence was found between the age groups (*p* = 0.042), but no significant difference was observed between genders (*p* = 0.236) (Table 1).

**Figure 4:**
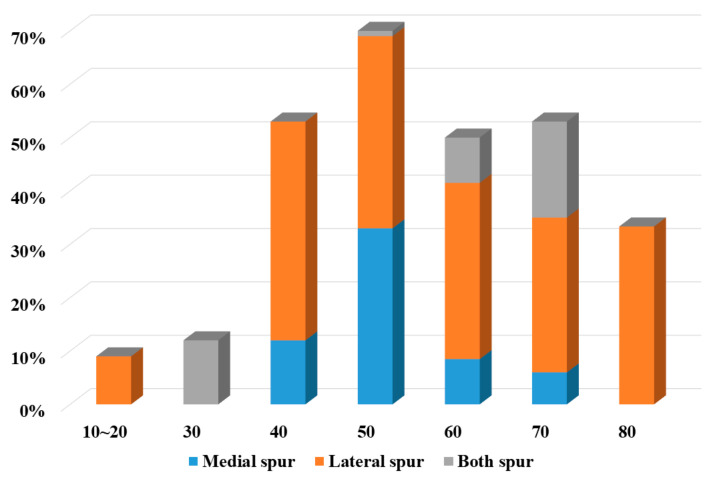
Cumulative distribution of biceps spur by spur type and age.

Medial spurs were identified in 13 cases (11.7%), lateral spurs in 37 cases (33.3%), and both spurs in 10 cases (9%). In Group I, 3 medial spurs (6.3%) and 17 lateral spurs (35.4%) were observed; while in Group II, 10 medial spurs (15.9%) and 20 lateral spurs (31.7%) were detected. Both spurs were found in two cases (4.2%) in Group I and in eight cases (12.7%) in Group II (Figure 4). No statistically significant differences were found in spur incidence by spur type.

Regarding gender, 8 medial spurs (13.6%) and 21 lateral spurs (35.6%) were observed in men; while in women, 5 medial spurs (9.6%) and 16 lateral spurs (30.8%) were identified. Both spurs were present in six men (10.2%) and four women (7.7%). Overall, spurs of all types were more frequent in men, and lateral spurs were more common than medial spurs in both genders. However, no statistically significant differences in spur incidence or type were found between genders.

In subgroup analysis, a statistically significant difference in spur incidence was found between the age groups in men (*p* = 0.042), with more spurs observed in the older age group. However, in women, no significant difference in spur types was observed between age groups (Table 2).

### 3.2. Anthropometry

The average width of the bicipital groove measured 11.8 ± 1.7 mm in Group I and 10.9 ± 1.6 mm in Group II. The average depth was 4.6 ± 0.7 mm in Group I and 4.6 ± 0.8 mm in Group II. The average medial wall angle was 58.9 ± 11.3° in Group I and 62.2 ± 10.1° in Group II. A statistically significant difference was found in the average width of the groove between the age groups (*p* = 0.006), with the elderly group showing a narrower groove. However, no significant differences were found in groove depth or medial wall angle between the age groups. The average height of the medial spur was 1.8 ± 0.7 mm in Group I and 1.7 ± 1.0 mm in Group II, while the average height of the lateral spur was 1.8 ± 0.6 mm in Group I and 1.9 ± 0.8 mm in Group II. No statistically significant differences were found in spur height between the age groups (Table 3).

The average width of the bicipital groove in men and women was 11.9 ± 1.6 mm and 10.6 ± 1.6 mm, respectively. The average depth was 4.9 ± 0.7 mm in men and 4.3 ± 0.6 mm in women. The average medial wall angle was 60.8 ± 10.7° in both men and women. Statistically significant differences were found in groove width and depth between genders, with men having larger measurements (*p* = 0.000 for both width and depth). The average height of the medial spur was 2.0 ± 0.9 mm in men and 1.1 ± 0.2 mm in women. The average height of the lateral spur was 1.8 ± 0.6 mm in men and 1.9 ± 0.8 mm in women. A statistically significant difference was found in the height of the medial spur between genders (*p* = 0.039) (Table 4).

The ICC values for width, depth, medial wall angle, height of the medial spur, and height of the lateral spur were 0.968, 0.841, 0.984, 0.975, and 0.933, respectively, indicating good to excellent reliability.

In the subgroup analysis within genders, in men, the width of the groove was narrower, while the depth, medial wall angle, and spur heights (both medial and lateral) were larger in patients older than 55 years. Statistically significant differences were found in the width, depth, and medial wall angle of the bicipital groove (*p* = 0.018, *p* = 0.012, and *p* = 0.044, respectively). In women, the measurements were similar between those younger and older than 55 years, with no statistically significant differences found (Table 5).

## 4. Discussion

This study demonstrated that 3D reconstructed images had a higher detection rate of bicipital spurs compared to 2D-CT axial images. Additionally, a greater number of spurs were observed in the elderly population. Although there are no previous reports, we found that the incidence of lateral spurs in the bicipital groove was higher than that of medial spurs. The bicipital groove was also found to be narrower in the elderly group. In terms of gender differences, the groove was wider and deeper in men compared to women.

Hitchcock et al. [4] described the ridges and spurs around the bicipital groove, suggesting that inflammatory changes originate from the ridge surrounding the bicipital groove. They proposed that these changes are supported by the presence of spurs on the lesser tuberosity. There have been several studies evaluating spurs around the bicipital groove, but their findings have been inconsistent. Farin et al. [8] examined 350 patients with ultrasonography and plain radiograph on bicipital groove. They observed 10 patients with osteophytes on plain radiograph and 2 patients with osteophytes on sonography. Pfahler et al. [17] examined 67 patients with ultrasonography and plain radiograph. They found two patients with osteophytes adjacent to the groove and three patients with osteophytes of the groove. Cone et al. [7] conducted an anatomical study describing medial wall and groove spurs using both cadaveric specimens and patient plain radiographs. In their study, medial spurs were found in 33% of cadaveric specimens and 17% of patients, while groove spurs were found in 20% of cadaveric specimens and 8% of patients. They attributed the difference in incidence to the higher quality of radiographs from cadaveric specimens, which allowed for the detection of smaller spurs that were not visible in patient radiographs. This is consistent with the findings of the present study, as both studies demonstrated a relatively high incidence of bicipital spurs and improved detection with methods that offer more intuitive visualization. Although the incidence of medial wall spurs (11.7%) and groove spurs (none) differed in this study, 3D reconstructed images allowed for clearer detection compared to 2D-CT axial images, similar to how Cone et al. achieved better detection in cadaveric specimens using radiographs. Additionally, this study observed a higher incidence of lateral spurs, a difference not examined in previous studies.

Meyer et al. [19] reported that medial spurs result from friction between the biceps tendon and the medial wall of the bicipital groove during internal rotation of the arm, while external rotation can lead to tendon attrition, contributing to the formation of lateral spurs. Cone et al. [7] suggested that the etiology of medial wall spurs is most likely traction by the transverse humeral ligament. Additionally, degenerative enthesopathy and the presence of bony excrescences on the floor of the bicipital groove may be related to chronic bicipital tenosynovitis. This aligns with the authors’ belief that spurs around the bicipital groove can irritate the long head of the biceps tendon during shoulder motion, potentially leading to biceps pathology.

The presence of medial and lateral bone spurs along the edges of the bicipital groove may have clinical implications for the long head of the biceps tendon (LHBT). Although Ricci et al. [20] did not specifically address bone spurs, they described the “hourglass” deformity of the LHBT, characterized by distal synovial sheath effusion due to gravity and proximal thickening within the rotator interval. This configuration can lead to intra-articular impingement during overhead arm movements, potentially causing chronic proximal tendinosis and restricted shoulder mobility. While our findings highlight the morphology of bicipital spurs, further research is needed to explore their role in LHBT pathology and the development of the hourglass deformity, which may inform treatment strategies for shoulder pathology associated with the biceps tendon.

There are few studies analyzing the clinical significance of the lateral wall, with Ueberham et al. [9] being one of the few to report an incidence of 32% for lateral spurs in a cadaveric study of the bicipital groove. In contrast, many studies on bicipital groove morphology emphasize the role of medial spurs. For instance, Urita et al. [21] found a strong association between medial biceps spurs and both subscapularis tears and LHBT disorders, suggesting a clinical link between medial spurs and shoulder pathology. However, Abboud et al. [16] reported that bicipital groove morphology, including medial wall angles, showed no significant correlation with intra-articular biceps pathology on MRI. These findings highlight the complexity of understanding biceps morphology and its clinical implications, indicating the need for further studies to clarify the roles of both medial and lateral spurs in shoulder conditions.

Radiologic signs of groove degeneration correlated with biceps tendon disease in 43.6% of cases using sonography [17]. This study also noted a higher incidence of LHB pathology in patients with a narrow bicipital groove, and a smaller medial wall angle was associated with LHB instability. However, other studies found no correlation between bicipital groove morphology on MRI and intraarticular biceps tendon pathology [16,20]. Therefore, in addition to our anatomical study, further research is needed to establish the relationship between bicipital groove morphology and biceps pathology.

The measurements in our study were consistent with those reported in the literature for width, depth, and medial wall angle [7,8,16,17,22,23]. In cadaveric studies [22,23], anthropometric measurements such as bicipital groove length and angle presented challenges. Studies using plain shoulder radiographs were limited in their ability to provide a 3D analysis of the bicipital groove [7,8,17]. In an ultrasound study [8], the measurements could vary since the diagnostic method is known to have limitations such as being user-dependent, prone to anisotropy, and having low reproducibility. In MRI, distinguishing biceps spurs from the transverse humeral ligament was difficult due to the similar low signal intensity of both structures [16]. Additionally, spurs may not be detected due to the thickness of the image slices, which can measure several millimeters. Since the cortex of bone is best visible on CT and 3D reconstructed image provides image in more fine thickness of slice with the scanned raw data, 3D-CT is suggested to be a more sensitive imaging method for detecting biceps spurs.

Although 3D-CT remains one of the most sensitive methods for detecting biceps spurs due to its high-resolution visualization of bony structures, other 3D imaging techniques, such as high-resolution 3D ultrasonography (HRUS), could serve as alternative modalities in specific clinical contexts. Recent research by Pušnik et al. [24] has highlighted the potential of 3D HRUS for anatomical evaluations and reconstructions in the musculoskeletal system, providing valuable non-invasive insights, especially in settings where a less invasive approach is preferable. Additionally, it can offer a cost-effective and accessible option for similar assessments.

This study had several limitations. First, the patients enrolled were not asymptomatic, as all had undergone shoulder CT scans for reasons related to shoulder joint pathology. Second, the study was retrospective and had a relatively small sample size. Third, the length and thickness of the humerus were not measured. However, a cadaveric study by Wafae et al. [20] found no correlation between the dimensions of the bicipital groove (width, depth, and length) and those of the humerus, suggesting that this limitation is unlikely to significantly affect our results. Finally, we did not analyze biceps pathologies, and further research is needed to explore the relationship between biceps spurs and biceps pathology.

The strength of our study lies in the fact that to our knowledge, this is the first report to analyze bicipital groove spurs using 3D-CT. Additionally, while reports on lateral spurs of the bicipital groove are scarce, our study includes an analysis of these spurs.

## 5. Conclusions

Using 3D-CT, the detection rate of bicipital spurs was higher than with 2D-CT. Bicipital groove spurs were more frequently observed in elderly individuals, and the groove width was narrower in this group. Men had a wider and deeper bicipital groove than women. Additionally, this study identified lateral spurs as the most common type of spur in the bicipital groove, a finding rarely reported in previous research.

## Figures and Tables

**Table 1:** Incidence of biceps spur.

	Group I (*n* = 48)	Group II (*n* = 63)	*p*-Value	Male (*n* = 59)	Female (*n* = 52)	*p*-Value
Total spur (%)	22 (45.8%)	38 (60.3%)	**0.014**	35 (59.3%)	25 (48.1%)	0.236
Medial spur (%)	3 (6.3%)	10 (15.9%)	0.145	8 (13.6%)	5 (9.6%)	0.519
Lateral spur (%)	17 (35.4%)	20 (31.7%)	0.684	21 (35.6%)	16 (30.8%)	0.591
Both spur (%)	2 (4.2%)	8 (12.7%)	0.182	6 (10.2%)	4 (7.7%)	0.748

Values in bold indicate statistical significance (*p* < 0.05).

**Table 2:** Subgroup analysis of incidence of biceps spur by gender.

Male	Under 55 (n = 35)	Over 55 (n = 24)	*p*-Value
Total spur (%)	17 (48.6%)	18 (75%)	**0.042**
Medial spur (%)	3 (8.6%)	5 (20.8%)	0.251
Lateral spur (%)	12 (34.3%)	9 (37.5%)	0.800
Both spur (%)	2 (5.7%)	4 (16.7%)	0.212
**Female**	**Under 55 (n = 13)**	**Over 55 (n = 39)**	***p*-Value**
Total spur (%)	5 (38.5%)	20 (51.3%)	0.423
Medial spur (%)	0 (0%)	5 (12.8%)	0.314
Lateral spur (%)	5 (38.5%)	11 (28.2%)	0.488
Both spur (%)	0 (0%)	4 (10.3%)	0.561

Values in bold indicate statistical significance (*p* < 0.05).

**Table 3:** Anthropometry of biceps groove and spur by age.

	Group I (n = 48)	Group II (n = 63)	*p* Value
Width (mm)	11.8 ± 1.7	10.9 ± 1.6	**0.006**
Depth (mm)	4.6 ± 0.7	4.6 ± 0.8	0.908
Medial wall angle (°)	58.9 ± 11.3	62.2 ± 10.1	0.103
Height of medial spur (mm)	1.8 ± 0.7	1.7 ± 1.0	0.627
Height of lateral spur (mm)	1.8 ± 0.6	1.9 ± 0.8	0.694

Values in bold indicate statistical significance (*p* < 0.05).

**Table 4:** Anthropometry of biceps groove and spur by gender.

	Male (n = 59)	Female (n = 52)	*p*-Value
Width (mm)	11.9 ± 1.6	10.6 ± 1.6	**0.000**
Depth (mm)	4.9 ± 0.7	4.3 ± 0.6	**0.000**
Medial wall angle (°)	60.8 ± 10.7	60.8 ± 10.7	0.990
Height of medial spur (mm)	2.0 ± 0.9	1.1 ± 0.2	**0.039**
Height of lateral spur (mm)	1.8 ± 0.6	1.9 ± 0.8	0.679

Values in bold indicate statistical significance (*p* < 0.05).

**Table 5:** Anthropometry of biceps groove and spur by gender.

Male	Under 55 (n = 35)	Over 55 (n = 24)	*p*-Value
Width (mm)	12.3 ± 1.5	11.4 ± 1.6	**0.018**
Depth (mm)	4.7 ± 0.7	5.2 ± 0.6	**0.012**
Medial wall angle (°)	58.4 ± 10.4	64.3 ± 10.5	**0.044**
Height of medial spur (mm)	1.8 ± 0.7	2.0 ± 1.0	0.734
Height of lateral spur (mm)	1.6 ± 0.5	1.9 ± 0.7	0.238
**Female**	**Under 55 (n = 13)**	**Over 55 (n = 39)**	***p*-Value**
Width (mm)	10.6 ± 1.6	10.6 ± 1.6	0.775
Depth (mm)	4.3 ± 0.6	4.3 ± 0.7	0.958
Medial wall angle (°)	60.2 ± 13.7	60.9 ± 9.8	0.627
Height of medial spur (mm)		1.1 ± 0.2	
Height of lateral spur (mm)	2.1 ± 0.7	1.9 ± 0.8	0.544

Values in bold indicate statistical significance (*p* < 0.05).

## Data Availability

The raw data supporting the conclusions of this article will be made available by the authors on request.

## Abbreviations

LHB: long head of biceps; CT: computed tomography; 3D: three-dimensional; 2D: two-dimensional.
